# Oral Health in a Sample of Pregnant Women from Northern Appalachia (2011–2015)

**DOI:** 10.1155/2015/469376

**Published:** 2015-05-18

**Authors:** Katherine Neiswanger, Daniel W. McNeil, Betsy Foxman, Manika Govil, Margaret E. Cooper, Robert J. Weyant, John R. Shaffer, Richard J. Crout, Hyagriv N. Simhan, Scott R. Beach, Stella Chapman, Jayme G. Zovko, Linda J. Brown, Stephen J. Strotmeyer, Jennifer L. Maurer, Mary L. Marazita

**Affiliations:** ^1^School of Dental Medicine, Department of Oral Biology, University of Pittsburgh, Bridgeside Point Suite 500, 100 Technology Drive, Pittsburgh, PA 15219, USA; ^2^Center for Oral Health Research in Appalachia (COHRA), University of Pittsburgh, Pittsburgh, PA 15219, USA; ^3^School of Dentistry, Department of Dental Practice & Rural Health, Eberly College of Arts and Sciences, Department of Psychology, West Virginia University, 53 Campus Drive, P.O. Box 6040, Morgantown, WV 26506, USA; ^4^School of Public Health, Department of Epidemiology, University of Michigan, 1415 Washington Heights, Ann Arbor, MI 48109, USA; ^5^School of Dental Medicine, Department of Dental Public Health, University of Pittsburgh, 3501 Terrace Street, Pittsburgh, PA 15213, USA; ^6^Graduate School of Public Health, Department of Human Genetics, University of Pittsburgh, 130 De Soto Street, Pittsburgh, PA 15261, USA; ^7^Department of Periodontics, West Virginia University, Room G110-B HSC, N. Medical Center Drive, Morgantown, WV 26506, USA; ^8^Department of Obstetrics, Gynecology and Reproductive Sciences, Magee-Womens Hospital, University of Pittsburgh Medical Center, 300 Halket Street, Pittsburgh, PA 15213, USA; ^9^University Center for Social and Urban Research, 3343 Fifth Avenue, Pittsburgh, PA 15213, USA

## Abstract

*Background*. Chronic poor oral health has a high prevalence in Appalachia, a large region in the eastern USA. The Center for Oral Health Research in Appalachia (COHRA) has been enrolling pregnant women and their babies since 2011 in the COHRA2 study of genetic, microbial, and environmental factors involved in oral health in Northern Appalachia. *Methods*. The COHRA2 protocol is presented in detail, including inclusion criteria (healthy, adult, pregnant, US Caucasian, English speaking, and nonimmunocompromised women), recruiting (two sites: Pittsburgh, Pennsylvania, and West Virginia, USA), assessments (demographic, medical, dental, psychosocial/behavioral, and oral microbial samples and DNA), timelines (longitudinal from pregnancy to young childhood), quality control, and retention rates. *Results*. Preliminary oral health and demographic data are presented in 727 pregnant women, half from the greater Pittsburgh region and half from West Virginia. Despite similar tooth brushing and flossing habits, COHRA2 women in West Virginia have significantly worse oral health than the Pittsburgh sample. Women from Pittsburgh are older and more educated and have less unemployment than the West Virginia sample. *Conclusions*. We observed different prevalence of oral health and demographic variables between pregnant women from West Virginia (primarily rural) and Pittsburgh (primarily urban). These observations suggest site-specific differences within Northern Appalachia that warrant future studies.

## 1. Introduction

**Figure 1:**
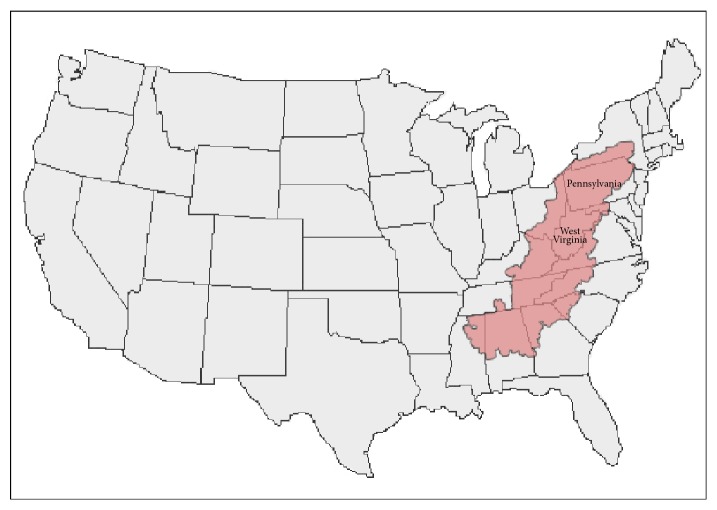
Appalachia (in pink, as defined by the Appalachian Regional Commission [1]) includes portions of twelve states, from New York in the north to Mississippi in the south. West Virginia lies completely within Appalachia, as does most of Pennsylvania, including Pittsburgh in the southwest corner.

Appalachia is a region of over 530,000 square kilometers in the eastern United States of America that roughly corresponds to the area surrounding the Appalachian Mountains [1]. It is largely rural and has been geographically isolated in the past because of mountainous terrain, as well as transportation systems that have hampered easy travel. Nevertheless, Appalachia is heterogeneous and includes urban areas: Pittsburgh, located in Allegheny county, Pennsylvania, is its largest city (2013 city population 306,000; county population 1,233,000 [2]).  Figure 1 shows the extent of Appalachia, as defined by the Appalachian Regional Commission, a partnership of federal, state, and local governments established in 1965 to promote community and economic development in the region [1].

Over 25 million people live in Appalachia. Historically, they have been burdened by relatively high levels of poverty. In 1960, for example, 31% of the population was living below the poverty level. This figure has declined to about 17% in the period 2009–2013, in comparison with 15.4% for the USA as a whole [1]. Nevertheless, in some parts of Appalachia, poverty and attendant issues such as food insecurity are quite prevalent. In 2013, for example, the per capita market income for Appalachia was almost US$10,000 less than the national average (US$27,979 versus US$37,127) [1].

Chronic poor oral health, with increased rates of dental caries, gingivitis, and periodontitis that can ultimately result in edentulism, has an unusually high prevalence in Appalachia [3]. In West Virginia, the only state located entirely within Appalachia, two-thirds of adults over 65 have lost 6 or more teeth and one-third are completely edentulous, compared to 40% and 17%, respectively, for the USA [4]. Caries rates in 16–19-year-olds are also increased in West Virginia over the general population (84% versus 67% [5, 6]). These oral health problems occur in the context of numerous other overall health issues (e.g., diet and nutrition, physical activity, obesity, diabetes, cardiovascular disease, and substance abuse) and are associated with a substantial regional health and economic burden [3].

Unlike many cultural differences in the USA that are defined by race and ethnicity, the geographical region also boasts a unique, yet heterogeneous, Appalachian identity [7], with its distinctive topography, foods, music, values, and behavior patterns. Sometimes characterized as a “neglected minority” [8], many of those living in rural Appalachia have been regarded as economically disadvantaged and undereducated. However, in spite of real and perceived health, economic, and social problems, people from Appalachia share numerous strengths, including independence, self-reliance, humility, modesty, sense of community and place, spirituality, patriotism, and (often self-effacing) humor [9].

The Center for Oral Health Research in Appalachia (COHRA) was established in 2000 as a collaboration between the University of Pittsburgh and West Virginia University to study the high rates of oral health problems seen in Northern Appalachia. Since then, COHRA has embarked upon multiple research programs to collect and study samples from this region. The first COHRA research program (COHRA1, 2000–2010) studied multiple genetic and environmental factors in families with children ages 1–18 living in central West Virginia and western Pennsylvania [10]. A high prevalence of caries was confirmed, even in the very young children in the study families. In particular, 5% of the COHRA1 two-year-olds, 21% of the three-year-olds, 35% of the four-year-olds, and 51% of the five-year-olds had some degree of decay. COHRA1 findings also suggest that individuals in Northern Appalachia have high rates of untreated caries even with increased use of sealants and have dental fear and anxiety that may be transmitted across generations [3].

In response to these findings, a second major study (COHRA2, 2011–present) was initiated to investigate oral health in pregnant women and their babies, specifically the factors predisposing a relatively high proportion of very young Northern Appalachian children to dental caries. COHRA2 was designed to test the hypothesis that the mechanisms leading to oral health disparities develop very early—within the first two years of life—and include a complex interplay among behavioral/environmental, microbiological, and genetic factors. To this end, COHRA2 is collecting extensive oral health, demographic, medical, dietary, behavioral, genetic, and microbiological data on a large sample of women and their babies from West Virginia and southwestern Pennsylvania over multiple time points and has reached 70% of its recruitment goal.

In this report, we describe the COHRA2 protocol and the oral health of the sample of COHRA2 pregnant women recruited from 2011 to 2015, including their oral health behaviors, personal and household demographics, and social behaviors.

## 2. Materials and Methods

**Figure 2:**
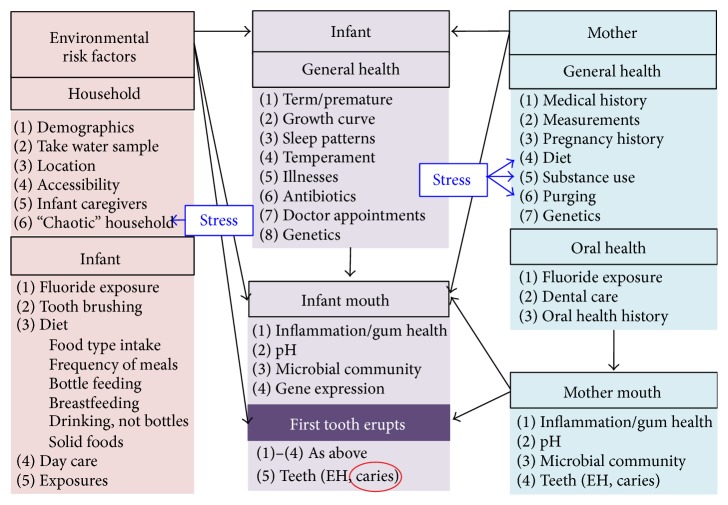
COHRA2 study design. Factors impacting infant caries (the outcome variable circled in red) are grouped into (1) environmental risk factors (pink), divided into household and individual risks external to the infant, (2) infant (purple), general health, which will influence infant's mouth, and (3) mother (blue), general and oral health, which may impact not only mother's mouth but also infant's general health and mouth. Mother's mouth may also directly affect infant's mouth. Stress may influence either the household or the mother directly. Infant's mouth is characterized before and after teeth erupt. Data are being collected for every numbered item in each category, as well as measures of stress. EH: enamel hypoplasia.

Under the COHRA2 study protocol, we collect data on women during their pregnancy and follow them and their babies longitudinally through the early years of the baby's life. As detailed in the COHRA2 study design (Figure 2), data are collected concerning the mother's general and oral health and the household and individual environment as well as the infant's general and oral health. Multiple microbial samples are taken from the mothers and babies at different time points, and human DNA is extracted from saliva. Several focused working groups (oral/dental health, medical history, diet, behavior, genetics, and microbiology) comprised of collaborators and outside experts designed the study. Protocol components are accomplished during several in-person visits and telephone interviews. Most in-person visits can be completed in an hour or less and telephone interviews in 45 minutes or less, minimizing participant burden and boosting retention.

### 2.1. Eligibility Criteria

Healthy US Caucasian women who are in the 12th to 29th weeks of pregnancy are potentially eligible for the study. In West Virginia, women who have not reached the 12th week of their pregnancy can be enrolled if necessary for scheduling appointments. Women must also be at least 18 years old, be relatively fluent in English, have a singleton pregnancy and cannot have tuberculosis or be immunocompromised. Women may also be excluded if they do not think they will remain in the general regions of West Virginia or southwestern Pennsylvania for the duration of the study, or if they do not have a reliable telephone contact. COHRA2 focuses on factors affecting normal healthy women and children, so if a woman delivers prematurely, that is, before the 35th week of her pregnancy, or if she or her baby develops a serious medical condition, they are withdrawn from the study. COHRA2 does not have an exclusion criterion based on baby's weight. If a low birth weight baby is delivered, COHRA2 consulting pediatric physicians determine on a case-by-case basis if there are additional health problems necessitating removal of the mother-baby pair from the study. Recruitment is limited to Caucasians to minimize the potential biases that ethnic heterogeneity can introduce into genetic analyses. Future studies are planned that will focus on women of different ethnicities from the same Northern Appalachian regions.

### 2.2. Sites

COHRA2 has two enrollment teams, one operating out of the University of Pittsburgh (Dr. Mary L. Marazita, co-PD/P.I.) and the other out of West Virginia University (Dr. Daniel W. McNeil, co-PD/P.I.), and a third team at the University of Michigan focusing on microbial ecology (Dr. Betsy Foxman, co-PD/P.I.). The Pittsburgh team recruits women who, for the most part, deliver at Magee-Womens Hospital of UPMC, Pittsburgh, PA, one of the largest hospitals in southwestern Pennsylvania with over 10,000 deliveries annually. In contrast, 20–22,000 babies are born annually in the entire state of West Virginia [5], necessitating statewide recruitment by the West Virginia team. The University of Michigan processes and analyzes the microbial samples from both recruitment sites. The University of Pittsburgh site is also the study coordinating center, receiving and analyzing data and samples from all sites.

### 2.3. Recruitment

Pittsburgh and West Virginia each plan to recruit at least 500 women, for a total initial sample size of over 1000 mother-baby pairs. In Pittsburgh, recruitment began in January 2012 and is being conducted primarily through the clinics and outreach offices of Magee-Womens Hospital. Flyers and brochures are distributed throughout the Magee health network and at other locations in the greater Pittsburgh area. On-site COHRA2 research staff and/or the Magee Clinical and Translational Research Center (CTRC) explain the study to potentially eligible women receiving prenatal care through physicians affiliated with Magee. Interested women provide their contact information, and the research staff contacts them with more details about the study. They are scheduled for an initial appointment before the end of their 29th week of pregnancy.

In West Virginia, recruitment began in November 2011, with women recruited throughout the state. Recruitment is facilitated with the help of 42 partnering health and dental clinics, health department offices, community centers, and hospitals. In addition, OB-GYN offices, WIC offices, Birth-To-Three offices, the West Virginia Perinatal Initiative, Early Head Start, and community service organizations recruit for the study. Print, radio, and television ads as well as brochures and posters are distributed periodically. The research team promotes the project through participation in health and pregnancy fairs, conferences, and interactive online presentations. Interested women contact the research staff, who screen for eligibility, provide details about the study, and schedule the initial appointments before the end of the 29th week of pregnancy.

The study has IRB approval from the University of Pittsburgh and West Virginia University. All potential participants have the study explained to them in detail and are sent copies of the consent forms before their initial appointments. At the first visit, the study is explained again, questions are answered, and the women sign consent forms prior to any research assessments.

### 2.4. In-Person Assessments

The in-person assessments consist of (1) an extensive dental assessment that includes collection of saliva and microbial samples (described in detail below); (2) physical measurements, including mother's and baby's height and weight, baby's head circumference, and mother's leg length [11, 12]; (3) an interview that includes basic demographics, pregnancy and medical history including antibiotic use, and questions specific to some time points; (4) an inventory of current medications; (5) the Dental Fear and Anxiety Scale, a 29-item scale that includes the 20-item Dental Fear Survey [13] plus the 9-item Fear of Pain Questionnaire [14]; (6) a portion of the Child Behavior Checklist [15]; and (7) a household water sample for determination of fluoride concentration. In Pittsburgh, 3D facial photographs and 2D hand scans are also taken to document growth parameters.

The dental assessment for mothers has several components, performed in the following order: (1) blood pressure measurements; (2) Oral Rating Index, a visual measure of gingival health and oral hygiene (ORI) [16]; (3) a modified version of the Whole Mouth Fluorosis Score [17]; (4) unstimulated salivary flow rate; (5) salivary pH; (6) microbial sampling of saliva, gingiva, and plaque; (7) soft tissue inspection; (8) malocclusion examination; (9) caries assessment; and (10) saliva collection for DNA extraction using Oragene·Discover kits (OGR-500, DNA Genotek).

Microbial samples are collected using OMNIgene**·**Discover kits (OM-501 or 505, DNA Genotek). Saliva is collected either by spitting or with swabs. Gingival swabs are taken from the maxillary and mandibular anterior buccal regions and the mandibular right posterior lingual region and pooled into an OMNIgene vial. Plaque is taken with a Stimudent or curette from three intact tooth surfaces (8-buccal, 24-buccal, 31-occlusal, or nearby surfaces if these are not intact) and pooled into an OMNIgene vial. Finally, plaque is taken from tooth surfaces with untreated lesions, including enamel hypoplasia, white spots, and cavitation of the enamel or dentin. Up to three surfaces presenting the same type of lesion are sampled and pooled. All sampled surfaces are recorded.

The caries assessment follows the PhenX Toolkit Dental Caries Experience Prevalence Protocol [18] (http://www.phenxtoolkit.org/, protocol number 080300), modified to be as simple as possible for evaluation of the dentition in two-year-olds. Tooth surface codes include a set of enamel hypoplasia codes following Oliveira et al. [19] and codes for active and inactive white spots. The tooth code for fluorosis has been replaced by the whole mouth fluorosis score [17], which facilitates the assessment of other tooth surface conditions. The modified tooth codes are compatible with the codes used in the COHRA1 study and allow decayed, missing, and filled tooth (DMFT) and surface (DMFS) scores to be calculated in two ways, including (D1MFT/S) and excluding (D2MFT/S) white spots.

The child dental assessment does not include the unstimulated salivary flow rate or the malocclusion exam and is greatly simplified when very few teeth have erupted. The Frankl Scale [20] rates the child's cooperativeness during the assessment.

The clinical protocol is performed by a licensed dentist or dental hygienist and a research assistant, using a dental chair with appropriate lighting. Participants are asked not to eat or brush their teeth for two hours before the dental assessment. Dental examiners and research assistants are trained in working with babies and young children, including use of the knee-to-knee position. The dental assessment does not include periodontal probing or any procedure that might induce bleeding. If women or children have oral health issues requiring treatment, appropriate referrals are made.

In Pittsburgh, research visits take place primarily at the University of Pittsburgh Center for Craniofacial and Dental Genetics (Dr. Mary L. Marazita, Director), with birth visits conducted at Magee-Womens Hospital of UPMC by either a member of the COHRA2 research staff or the Magee CTRC nurses. The research suite of the CCDG includes two fully equipped dental cubicles plus additional rooms for interviews and physical measurements. In West Virginia, assessments are performed by the research staff at the two main study coordinating sites in Morgantown and Summersville, which have fully equipped dental cubicles, as well as at the 42 statewide partner sites described above. In practice, some of the more remote sites are not utilized often, because it is not cost-effective for the research team to drive several hours for a single appointment for which a participant may not show.

### 2.5. Telephone Questionnaires

Most of the demographic and behavioral data is collected via a telephone questionnaire that is administered multiple times to the mothers from both sites by the University of Pittsburgh Center for Social and Urban Research (UCSUR, Dr. Scott R. Beach, co-I; http://ucsur.pitt.edu/). This 30–45-minute interview collects data on mother and baby, including demographics (education, ethnicity, household composition, medical and dental insurance, and income), detailed food and beverage intake over the past week, dietary habits (frequency of meals, food anxiety, and purging), breastfeeding and bottle feeding routines, oral hygiene, medical and dental histories (developmental delays, hospitalizations, and use of dental services), social behaviors and exposures (smoking, alcohol, and recreational drug use), the Center for Epidemiologic Studies Depression scale (CES-D) [21], the Perceived Stress Scale (10-item PSS) [22], a single omnibus “overall fear” item from the Dental Fear Survey [13], caregivers inside and outside the home, and baby's temperament, tooth eruption history, and sleeping habits, including the Brief Infant Sleep Questionnaire (BISQ) [23]. If women score 27 or higher on the CES-D scale, they are sent a letter advising that they may have symptoms of depression, along with a list of mental health resources including contact information specific to their place of residence.

### 2.6. Longitudinal Study Timeline

As summarized in Table 1, participants complete in-person assessments at 4–6 time points: (1) prenatal visit (between 12 and 29 weeks of pregnancy); (2) birth visit (Magee-Womens Hospital, Pittsburgh only); (3) pretooth visit (baby is 2-3 months old); (4) first-tooth visit (one month after the eruption of baby's first tooth, Pittsburgh only); (5) 1-year visit; and (6) 2-year visit. The UCSUR telephone questionnaire is administered once prenatally and five times postnatally—about every six months through the course of the study. In the future, this timeline may be extended to older children.

Not all parts of the protocol are performed at every visit (Table 1). In particular, the birth visit (Pittsburgh only) has an abbreviated protocol and serves mainly to collect microbial samples before baby goes home from the hospital. Pittsburgh also performs an in-person assessment within one month after the eruption of baby's first tooth, in order to compare baby's microbial community before and shortly after a tooth erupts. The research team in Pittsburgh conducts a monthly Short Phone Interview (SPI) that includes questions about breastfeeding, baby's diet and beverage intake, health issues, including doctor visits and antibiotic use, and any tooth eruptions. The purpose of the SPI is to get baseline information on a frequent basis and to schedule the first-tooth visit. After the first tooth erupts, the SPI is performed every 2-3 months. Logistical issues involving extensive travel to research facilities statewide prevent the West Virginia team from conducting the birth visit, first-tooth visit, and the SPI.

The telephone interview is conducted six times—once prenatally and five times postnatally, when the baby is about 10 weeks old, 6 months old, 12 months old, 18 months old, and 24 months old.

### 2.7. Quality Control

Data quality is maintained through multiple mechanisms. By utilizing UCSUR, the uniformity and quality of the questionnaire data are maintained across sites, freeing site research staff to focus on recruiting, scheduling, and conducting study appointments. COHRA2 has an external review board—Drs. John J. Warren, Teresa A. Marshall, and Jeff Murray—who have contributed their expertise in pediatric dental research, dietary assessments, and genetics, respectively. Oversight is provided by the NIDCR through a Clinical Study Oversight Committee (CSOC) that monitors scientific issues and progress in sample acquisition and periodic site visits by the Clinical Research Operations and Management Support (CROMS) system.

### 2.8. Training/Calibration

All members of the Pittsburgh and West Virginia research teams are trained and certified in the conduct of human subject research (CITI modules). The dental examiners have been trained and calibrated on the tooth codes from the caries assessment on a regular basis since 2011, before data collection began. West Virginia examiners conducted an initial training/calibration in March 2012, at which 10 adults were assessed by four raters twice on two consecutive days. On day 1, interrater reliability scores (Cohen's* kappa*) for sound, decayed, and filled groups of tooth surface codes ranged from 71.8 to 86.1. Following training and analysis of day 1,* kappas* improved to 82.1–92.5, indicating substantial to excellent agreement.

Formal training/calibration sessions are conducted on a regular basis for examiners from all sites, using adults, older children, and COHRA2 participants. Staff are compared to a “gold standard” dental examiner, following the guidelines of the Early Childhood Caries Collaborative Centers (EC4, http://oralhealthdisparities.ucsf.edu/), which was provided by Dr. John J. Warren (Protocol for the Training and Calibration of Dental Examiners, unpublished) during a two-day training and calibration session in Pittsburgh, PA. To calibrate raters, tooth surface codes are collapsed into two categories—“sound” and “decayed/filled” teeth—which are necessary to calculate DMFT scores. Cohen's* kappas* are calculated to determine the reliability of this distinction. By this measure, the two most experienced raters (JGZ, U Pitt; LB, WVU) have an excellent* kappa* of 87.8, ensuring good reliability across sites and making it possible for both of them to serve as gold standards for training and calibrating newer assessors at each site. In general, the average* kappa* across all staff (compared to JGZ) is 70.8, indicating substantial agreement.* Kappas* for individual staff members range from 46.1 to 80.6. During a calibration session, discrepant tooth scores are discussed immediately, so that initially moderate agreement is improved at subsequent sessions.

For the telephone questionnaires administered by UCSUR, all telephone interviewers are trained in general survey interviewing techniques via a standard three-day UCSUR protocol and receive project-specific training, including detailed question-by-question instructions, from the Pittsburgh site investigators.

### 2.9. Statistical Analyses

Raw data for categorical variables were collapsed for cells with very small numbers, and missing/unknown responses (less than 1% unless noted) were removed. Since several women completed their prenatal in-person visit but were withdrawn before their prenatal telephone interview, the total available responses differed for different variables. Data were analyzed using the R statistical environment (R Foundation for Statistical Computing, Vienna, AU). Means for three variables (age, D1MFT, and D2MFT) were compared using the nonparametric Wilcoxon-Mann-Whitney test due to significantly nonnormal distributions. Comparisons of categorical variables were performed using the chi-square test with appropriate degrees of freedom, with the Yates continuity correction for 2 × 2 tables. Fisher's exact test was employed in the event that any set of data for a categorical variable did not satisfy the conditions for the chi-square test.

## 3. Results

### 3.1. Sample Retention

As of January 31, 2015, the COHRA2 study has enrolled 744 pregnant women, 368 from Pittsburgh and 376 from West Virginia. Seventeen of these women have been enrolled twice, during two separate pregnancies, in order to conduct preliminary microbiological studies of siblings, so 727 independent women have completed the first prenatal in-person visit. Of these 727 women, 153 subsequently have been withdrawn at different stages of the protocol (52 from Pittsburgh, 101 from West Virginia), for an overall retention rate of 79% (574/727). There are several reasons that mother-baby pairs became ineligible. First, premature delivery (less than 35 weeks' gestational age) resulted in 13 women being withdrawn (8 from Pittsburgh, 5 from West Virginia), for a premature birth rate of 1.8% (13/727). Other reasons participants became ineligible include babies and/or mothers developing serious health problems (*n* = 8), mothers losing custody or not living with their children (*n* = 8), mothers moving out of the region (*n* = 6), baby deaths (*n* = 5), and miscarriages (*n* = 2), for a total of 42 women withdrawn because they became ineligible. The remaining 111 withdrawn women were lost to follow-up.

### 3.2. Sample Characteristics

Summary statistics for the 727 women are provided in Tables 2–6. These data were taken from the first in-person assessment and the first telephone interview, that is, during pregnancy, and divided by site. Data from the 153 women who later were withdrawn are included in these tables. Tables 2 and 3 summarize the women's oral health status and behaviors, respectively. Caries status is summarized in Table 2 by the D1MFT and D2MFT scores, which differ based on the inclusion or exclusion of white spots. Both DMFT scores are significantly elevated in West Virginia, compared to Pittsburgh, indicating higher rates of caries in West Virginia. A high percentage of women in both sites have some degree of decay. Gingival health is documented in Table 2 by the ORI score and a self-reported frequency of gingival bleeding during tooth brushing. Both of these measures suggest significantly better gingival health in the Pittsburgh sample. In particular, 75% of the women in Pittsburgh have ORI scores of excellent or good, compared to 55% of the women from West Virginia.

Tooth brushing and dental flossing habits are roughly the same between sites (Table 3). Over 70% of the women at both sites brush their teeth at least twice a day. Dental flossing habits are slightly different. More women sampled in Pittsburgh floss (75% versus 67%, *P* = 0.02), but they floss less frequently than the flossers in West Virginia (*P* = 0.03). Women in West Virginia visit the dentist less frequently than women in Pittsburgh (*P* < 0.00001). For example, 79% of the Pittsburgh sample reported a visit to the dentist within one year, compared to 57% of the West Virginian women.

Table 4 provides personal demographic statistics for the pregnant women. Approximately 38% of the women in the study are first-time moms, and 97% of the women have some type of medical insurance. Women in the West Virginia sample are significantly younger than the Pittsburgh sample (average age 27.0 years versus 29.8 years, *P* < 0.00001). They have significantly less education and higher rates of unemployment, and fewer of them have dental insurance (42% versus 87%, *P* < 0.00001). Finally, more of the West Virginian sample self-reports their general health to be fair or poor, compared to Pittsburgh.

Household demographics are provided in Table 5. About 5% of the women are living by themselves. For the women living with at least one other person, the households in West Virginia are significantly larger, due to increased numbers of children in the household (*P* = 0.0002). Women in West Virginia live in households that have significantly lower income (*P* < 0.00001) and significantly more anxiety about food. 23% of households in the West Virginia sample occasionally or often ran out of food in the past year, versus 12% from Pittsburgh (*P* = 0.001).

Alcohol use and smoking habits are summarized in Table 6. Significantly more pregnant women in the Pittsburgh sample reported drinking in the period beginning three months prior to the pregnancy and continuing into the second trimester (75% versus 53%, *P* < 0.00001), while more women in the West Virginia sample reported smoking (42% versus 32%, *P* = 0.02). Among the women who drank immediately before the pregnancy, a higher percentage in West Virginia stopped drinking in the first trimester (73% versus 61%, *P* = 0.01) and the second trimester (94% versus 84%, *P* = 0.005). With regard to smoking, 23% of the smokers at both sites did not smoke in the first trimester of pregnancy, and about 46% did not smoke in the second trimester.

## 4. Discussion

Caries develops from an imbalance between demineralization and remineralization of tooth surfaces, when diet, oral hygiene, and the intrinsic features of the oral cavity create an environment favorable for cariogenic bacteria [24]. In addition to the direct pathogenesis, however, multiple components operating on individual, family, and community levels influence caries risk [24–26], including factors that are specific to certain geographical regions [3] or stages of life, such as pregnancy or young childhood [27–30].

The COHRA2 study is examining many of these factors in pregnant women and their babies over the early years of life, sampled from West Virginia and the greater Pittsburgh region. Here, we describe the protocol and present summary data from 727 pregnant women enrolled to date. Women who delivered at a gestational age of 35-36 weeks were retained in the study, even though the standard definition of full term delivery begins at 37 weeks. Review of the literature determined that a very high percentage of babies born at gestational ages of 35-36 weeks are healthy, allowing us to retain these women and resulting in relatively low exclusion rates based on premature delivery. Sample collection is ongoing; thus, these are preliminary data.

We observe that our samples of pregnant women recruited into COHRA2 in West Virginia, when compared to the sample recruited in Pittsburgh, are younger, have worse oral health and similar brushing and flossing habits, see the dentist less often and have less dental insurance, less education, and more unemployment, live in households with more children and less income, drink less, and smoke more. Additional work by the COHRA2 research team relates pregnant women's depression status to site differences [31], with possible clinical implications for dental practitioners.

Recruiting practices between the Pittsburgh and West Virginia sites may account for some of these observations. Pittsburgh recruits in an urban setting through a large women's hospital. Pregnant women are approached about the study and asked if they are interested; only a few answer more general ads or respond to brochures. In contrast, there is no centralized source of pregnant women in largely rural West Virginia, and a statewide health partnership network has been built, along with extensive advertising, to facilitate recruiting. These divergent recruiting strategies—although necessary given the nature of the populations—may result in different types of samples and may reflect an overall urban/rural difference between the two sites.

*Limitations*. The COHRA2 study is still in the process of recruiting participants; thus, sample sizes will be larger in the future. However, with 70% of the target sample size completed, the sample is sufficiently large for initial description of the data. Increasing the sample should increase power but not fundamentally alter the nature of the observations presented in Tables 2–6. Nonetheless, these data should be considered preliminary.

More fundamentally, these observations cannot address causation of the differences between sites. The COHRA2 study was not designed to investigate differences between women sampled in West Virginia versus Pittsburgh. Both Pittsburgh and West Virginia are part of Northern Appalachia, where oral health disparities are known to occur. Pittsburgh, located about 120 kilometers from West Virginia University in Morgantown, has some of the same geographical qualities that are characteristic of West Virginia, that is, a mountainous terrain broken up by river valleys. However, the observed demographic differences between subjects enrolled in the Pittsburgh and West Virginia sites reveal that Northern Appalachia is not one large demographically homogeneous region and serve to validate the design of the COHRA2 study in selecting sites from different regions in Northern Appalachia. These demographic differences also underscore the importance of stratifying by site when studying genetic, environmental, and microbial factors contributing to poor oral health in the Appalachian region.

## 5. Conclusions

Samples of pregnant women from the COHRA2 study in West Virginia and Pittsburgh show different levels of oral health problems and have several different demographic properties. However, as the COHRA2 study looks for associations between risk factors and dental caries, including the interactions between genetics, microbiology, and diet and other exposures, the associations should be more broadly generalizable, even if the prevalence of these factors is specific to the two study sites. The COHRA2 study provides an invaluable wealth of data for understanding the oral health issues facing this region.

## Figures and Tables

**Table 1:** Timeline of COHRA2 in-person assessments.

Assessment	In-person visits					
	1 (prenatal)	2^1^ (birth)	3 (2-month)	4^1^ (first tooth)	5 (12-month)	6 (24-month)
Dental assessment	M^2^	M, B^2^	M, B	M, B	M, B	M, B
Physical measurements^3^	M	B	M, B	M, B	M, B	M, B
Medical interview	M^4^	M^5^	M	M	M	M
Current medications	M	M, B	M, B	M, B	M, B	M, B
Dental Fear Survey	M					
Fear of Pain Questionnaire	M					
Child Behavior Checklist (partial)						M
Household water^6^	M					
Microbial samples						
Gingival swab	M	M, B	M, B	M, B	M, B	M, B
Saliva	M	M	M, B	M, B	M, B	M, B
Tooth plaque	M	M	M	M, B	M, B	M, B
Saliva for DNA^7^	M		B			

^1^Visit performed at Pittsburgh site only.

^2^M: mother; B: baby.

^3^Not all measurements performed at all visits; self-reported measurements recorded if physical measurements are unable to be made.

^4^Questions on preterm labor included, to insure that participant can safely complete protocol.

^5^Birth information collected by phone if birth visit is not completed.

^6^Collected at visit 1; if participant moves, additional sample is collected from new residence.

^7^Saliva collected at additional visits as needed to insure adequate amounts of DNA.

**Table 2:** Oral health of COHRA2 women during pregnancy (2011–2015).

Variable	Pittsburgh		West Virginia		Combined		*P* value^1^
	*N*	Mean (±SD)	*N*	Mean (±SD)	*N*	Mean (±SD)	
*Dental caries *							
Average D1MFT^2^	357	8.86 (±7.3)	363	13.47 (±7.3)	720	11.18 (±7.6)	<0.00001^3^
Average D2MFT^2^	357	6.71 (±5.7)	363	7.89 (±6.1)	720	7.30 (±5.9)	0.007^3^

	*N*	%	*N*	%	*N*	%	*P* value

D1MFT							
0	27	8%	2	1%	29	4%	<0.00001
>0	330	92%	361	99%	691	96%	
D2MFT							
0	40	11%	24	7%	64	9%	0.04
>0	317	89%	339	93%	656	91%	
*Gingival health *							
Mouth bleeding							
Does your mouth bleed when you brush?							
Yes	124	37%	153	48%	277	42%	0.004
No	213	63%	164	52%	377	58%	
If yes, how often?							
≤2-3 times a month	35	28%	42	28%	77	28%	0.10
1 time a week	19	15%	27	18%	46	17%	
2–6 times a week	40	32%	31	20%	71	26%	
≥1 time a day	30	24%	52	34%	82	30%	
ORI score^4^							
Excellent	72	20%	54	15%	126	18%	<0.00001^5^
Good	194	55%	146	40%	340	47%	
Questionable	1	0%	7	2%	8	1%	
Poor	65	18%	126	35%	191	27%	
Very poor	23	6%	29	8%	52	7%	

^1^ *P* value tests differences between Pittsburgh and West Virginia sites.

^2^D1MFT: decayed, filled, and missing tooth score, white spots coded as decay; D2MFT: decayed, filled, and missing tooth score, white spots coded as sound.

^3^Wilcoxon rank sum test with continuity correction.

^4^ORI, measure of gingival health.

^5^Fisher exact test.

**Table 3:** Oral health behaviors of COHRA2 women during pregnancy (2011–2015).

Variable	Pittsburgh		West Virginia		Combined		*P* value^1^
	*N*	%	*N*	%	*N*	%	
*Tooth brushing* ^2^							
How often do your brush your teeth?							
<1 time a day	11	3%	10	3%	21	3%	0.56
1 time a day	72	21%	82	26%	154	23%	
2 times a day	221	65%	193	61%	414	63%	
>2 times a day	34	10%	34	11%	68	10%	
*Dental flossing *							
Do you floss?							
Yes	255	75%	213	67%	468	71%	0.02
No	84	25%	106	33%	190	29%	
If yes, how often?							0.03
≤1 time a week	69	27%	60	28%	129	28%	
2–6 times a week	93	36%	65	31%	158	34%	
1 time a day	77	30%	58	27%	135	29%	
>1 time a day	16	6%	30	14%	46	10%	
*Dental visits* ^3^							
How long since last visit?							<0.00001
<1 year	262	79%	177	57%	439	68%	
1-2 years	31	9%	57	19%	88	14%	
>2 years	40	12%	74	24%	114	18%	

^1^ *P* value tests differences between Pittsburgh and West Virginia sites.

^2^100% of women report that they brush their teeth.

^3^98% of women have been to a dental practitioner.

**Table 4:** Personal demographics of COHRA2 women during pregnancy (2011–2015).

Variable	Pittsburgh		West Virginia		Combined		*P* value^1^
	*N*	Mean (±SD)	*N*	Mean (±SD)	*N*	Mean (±SD)	
Average age	357	29.8 (±5.1)	366	27.0 (±5.4)	723	28.4 (±5.4)	<0.00001^2^

	*N*	%	*N*	%	*N*	%	*P* value

Education							
<High school degree	13	4%	37	10%	50	7%	<0.00001
High school degree	37	10%	94	26%	131	18%	
Some college	101	28%	130	36%	231	32%	
College degree	105	29%	64	17%	169	23%	
Degree beyond college	102	28%	41	11%	143	20%	
*Employment *							
Ever employed?							
No	5	1%	24	7%	29	4%	0.0008
Yes	353	99%	342	93%	695	96%	
If yes, current employment status							
Unemployed	79	22%	150	44%	229	33%	<0.00001
Part time employment	64	18%	67	20%	131	19%	
Full time employment	178	50%	109	32%	287	41%	
Student/other	32	9%	16	5%	48	7%	
Prior pregnancies							
0	146	41%	129	35%	275	38%	0.15
1+	213	59%	237	65%	450	62%	
Type of medical insurance^3^							
Private/through employer	216	65%	127	42%	343	54%	<0.00001
Medicare	9	3%	16	5%	25	4%	
Medical assistance	75	23%	27	9%	102	16%	
Medicaid	20	6%	119	39%	139	22%	
Other	12	4%	16	5%	28	4%	
*Dental insurance *							
Do you have dental insurance?							
Yes	289	87%	128	42%	417	65%	<0.00001
No	42	13%	179	58%	221	35%	
If yes, what type?							
Private/through employer	192	66%	106	83%	298	71%	0.001
Other	97	34%	22	17%	119	29%	
Self-report general health^4^							
Excellent	103	29%	74	20%	177	25%	0.002
Good	214	60%	223	61%	437	61%	
Fair or poor	39	11%	68	19%	107	15%	

^1^ *P* value tests differences between Pittsburgh and West Virginia sites.

^2^Wilcoxon rank sum test with continuity correction.

^3^97% of women have medical insurance.

^4^“Compared to other women your age, how would you rate your general health?”

**Table 5:** Household demographics of COHRA2 women during pregnancy (2011–2015).

Variable	Pittsburgh		West Virginia		Combined		*P* value^1^
	*N*	%	*N*	%	*N*	%	
Additional people in household							
0	22	6%	13	4%	35	5%	0.002
1	153	45%	109	34%	262	39%	
2+	168	49%	201	62%	369	55%	
*For households with additional people *							
Additional adults							
0	15	5%	17	5%	32	5%	0.48
1	261	81%	240	77%	501	79%	
2+	45	14%	53	17%	98	16%	
Additional children							
0	185	56%	128	41%	313	49%	0.0002
1	86	26%	99	32%	185	29%	
2+	58	18%	87	28%	145	23%	
Household income (US$)^2^							
<10,000	24	8%	43	15%	67	11%	<0.00001
10,000–24,999	36	12%	67	24%	103	17%	
25,000–49,999	49	16%	75	27%	124	21%	
50,000–99,999	117	38%	68	24%	185	31%	
100,000+	86	28%	29	10%	115	19%	
*Food anxiety in past year *							
Worried food might run out							
Never true	283	83%	227	71%	510	77%	0.001
Sometimes true	47	14%	74	23%	121	18%	
Often true	10	3%	18	6%	28	4%	
Food did run out							
Never true	298	88%	247	77%	545	83%	0.001
Sometimes true	33	10%	57	18%	90	14%	
Often true	7	2%	15	5%	22	3%	

^1^ *P* value tests differences between Pittsburgh and West Virginia sites.

^2^7% unknown/refused.

**Table 6:** Social behaviors of COHRA2 women during pregnancy (2011–2015).

Variable	Pittsburgh		West Virginia		Combined		*P* value^1^
	*N*	%	*N*	%	*N*	%	
*Alcohol use *							
Did you drink from 3 months prior to pregnancy through 2nd trimester?							
Yes	254	75%	170	53%	424	64%	<0.00001
No	86	25%	149	47%	235	36%	
If yes, number who drank^2^							
3 months prior to pregnancy	252	99%	163	96%	415	98%	ND^3^
1st trimester	98	39%	45	27%	143	34%	0.01
2nd trimester	40	16%	10	6%	50	12%	0.005
*Smoking *							
Did you smoke from 3 months prior to pregnancy through 2nd trimester?							
Yes	110	32%	133	42%	243	37%	0.02
No	230	68%	186	58%	416	63%	
If yes, number who smoked^2^							
3 months prior to pregnancy	110	100%	129	97%	239	98%	ND
1st trimester	85	77%	102	77%	187	77%	1.00
2nd trimester	62	56%	65	52%	127	54%	0.59

^1^ *P* value tests differences between Pittsburgh and West Virginia sites.

^2^Totals for these tests are equal to or slightly less than the total who responded “yes.”

^3^ND: not done.
